# Early Gastric Cancers in Central Norway 2001 to 2016—A Population-Based Study

**DOI:** 10.3390/cancers16061222

**Published:** 2024-03-20

**Authors:** Camilla J. Kvamme, Thomas L. Stillingen, Alina D. Sandø, Patricia Mjønes, Erling A. Bringeland, Reidar Fossmark

**Affiliations:** 1 Department of Clinical and Molecular Medicine, Faculty of Medicine, Norwegian University of Science and Technology (NTNU), 7030 Trondheim, Norway; camillajoerdrekvamme@gmail.com (C.J.K.); alina.desiree.sando@stolav.no (A.D.S.); patricia.mjones@stolav.no (P.M.); erling.bringeland@stolav.no (E.A.B.); 2 Department of Gastrointestinal Surgery, St. Olav’s Hospital, Trondheim University Hospital, 7030 Trondheim, Norway; 3 Department of Pathology, St. Olav’s Hospital, Trondheim University Hospital, 7030 Trondheim, Norway; 4 Department of Gastroenterology, St. Olav’s Hospital, Trondheim University Hospital, 7030 Trondheim, Norway

**Keywords:** gastric cancer, early gastric cancer, epidemiology, surgery, survival

## Abstract

**Simple Summary:**

Stomach cancer is rarely diagnosed at early stages, while it is still easy to cure. We gathered information from national registries and evaluated all patients with early stomach cancer in a defined healthcare region and re-examined tissue samples to characterize this group. We found that only 88 of 1205 stomach cancers from 2001 to 2016 were early cancers, with no major change in how common early cancers were during the 15-year study period. Specific features of the early gastric cancer (EGC), such as size, growth depth, growth into veins, and ulceration, were associated with spreading to regional lymph nodes. The patients were followed up for a minimum of five years after the operation, and most died of causes unrelated to stomach cancer. However, 12.7% of the deaths were cancer-related, with similar proportions being due to complications after surgery and recurrence of EGC, which could manifest itself more than five years after diagnosis.

**Abstract:**

Early gastric cancers (EGCs) are confined to the gastric mucosa and submucosa irrespective of lymph node metastases and constitute only a minor proportion of gastric cancer in Western countries. We aimed to characterize EGCs and assess the survival of EGC in Central Norway during 2001–2016. A retrospective population-based study on 1205 patients with gastric cancer was performed. At the time, surgical resection was the standard treatment, and 88 (7.3%) EGCs were identified. Histopathological specimens were re-examined, and the eCura score and survival were evaluated. The number of gastric cancers declined (*p* = 0.010), but the relative proportion of EGC was unchanged during the study period. EGCs were more often of the Lauren intestinal type (*p* < 0.001) compared with controls. A significant proportion (9.4%, n = 5) of the patients with a low-risk eCura had lymph node metastases, whereas further exclusion of tumors with histological ulceration or SM2 invasion identified an N0 cohort. The median survival for EGC patients was 117.1 months (95% CI 99.8–134.3) and the 5-year overall survival was 75%. Twelve deaths were cancer-related, either due to postoperative complications (5.7%, n = 5) or cancer recurrence (8%, n = 7). In conclusion, EGCs constituted a minor but constant proportion of gastric cancers. eCura alone was insufficient in predicting patients with pN0 disease.

## 1. Introduction

Gastric adenocarcinoma is the world’s fifth most common cancer and the fifth leading cause of cancer-related mortality [1]. The age-adjusted annual incidence of gastric adenocarcinoma in Norway declined from above 40/100.000 to below 6/100.000 over the past 60 years [2]. Reduced prevalence of *Helicobacter pylori* and gastric cancers of the Lauren histological intestinal type is the dominant cause for this reduction in Western populations [3,4]. However, the Lauren diffuse-type cancers [4], non-cardia gastric cancers in younger age cohorts, and cancers located in the gastric corpus and fundus [5] have increased in the United States. The five-year relative survival of patients with gastric cancer in Norway has improved from 17.3% to 27.4% over the past 30 years [2]. This is explained mainly by a marked increase in the survival of patients with localized or regional disease [2], contingent on improved surgery and the introduction of perioperative chemotherapy as national standard of care in 2007 [6]. Tumor resection is the cornerstone in curative treatment, but unfortunately, at a population level, surgery has a curative potential only in a minor proportion of the patients [7]. Given the marked association between disease stage and survival [8], the diagnosis of gastric cancers at an earlier stage is wanted.

The proportion of gastric cancers diagnosed at an early stage varies considerably between Western countries [9], where no general screening programs for gastric cancer have been implemented [10]. Early gastric cancer (EGC) is a technical term defined as a malignancy confined to the gastric mucosa and submucosa irrespective of lymph node metastases (LNM) [11,12], and it is an infrequent finding in Western registries. The diagnostic criteria for EGC differ between Japan, where criteria are based on cellular or structural atypia irrespective of invasion trough the lamina propria, and the West, where criteria emphasize penetration of neoplastic tissue beyond the lamina propria [11,13]. Studies of EGC must therefore be interpreted with caution across classification systems [14], and reports restricted to Western cohorts has a value of its own both to endoscopists and surgeons. Until the last decade, the majority of EGC patients in many European countries were treated by formal surgical resection, including standard lymphadenectomy. However, endoscopic submucosal dissection (ESD) enables en bloc resection of the tumor and accurate histopathologic risk stratification and is curative in a large proportion of patients with EGC. Patients with EGC treated with ESD within traditional Japanese criteria have a very low risk of lymph node metastases [15], also when using the more recent expanded criteria [16]. Tumors that are post hoc histologically proven to be outside criteria for curative ESD have a risk of lymph node metastases of >1% and should be considered for rescue gastrectomy with adequate lymphadenectomy after ESD, depending on age and comorbidity [17,18,19]. Hence, evaluating the adequacy of the ESD selection criteria by examining the surgical EGC specimens and long-term survival rates is valuable. The aim of the present study was to characterize EGCs in Central Norway before ESD was an available treatment option. Furthermore, we aimed to assess histopathological features associated with lymph node metastases and long-term survival patterns in our EGC patient cohort.

## 2. Materials and Methods

### 2.1. Study Design and Data Source

This study was a retrospective population-based study using a database described in previous publications [7,20,21]. A total of 1205 patients were diagnosed with gastric adenocarcinoma in Central Norway between January 2001 and December 2016, including tumors at the gastric cardia, Siewert types II/III. The time-averaged catchment area of approximately 680,000 persons comprised 15% of the Norwegian population during the study period. Patients were identified from the Norwegian Cancer Registry as well as from the Norwegian Patient Registry (NPR), and the diagnosis was validated and individual data extracted by a review of the medical records for each patient. Clinical and histopathological characteristics were recorded using the methodology previously described [7,20,21]. The annual numbers of gastroscopies performed in Norway in the study period were provided by the NPR and served as a proxy for the development in Central Norway.

### 2.2. Early Gastric Cancer and Control Groups

Early gastric cancer was defined as a malignancy confined to the gastric mucosa or submucosa (pT1) irrespective of lymph node metastases. Patients with carcinoma in situ were not included in the study, in accordance with Western diagnostic criteria for early gastric cancer [22]. Patients who received neoadjuvant chemotherapy and were histologically staged to ypT0 or ypT1 following surgery were also excluded in order to study only chemo-naïve (true) EGC. The standard procedure for EGC treatment during the entire study period was gastrectomy with a modified D2 resection. In a sub-analysis, EGCs were subdivided based on the endoscopic curability (eCura) score risk stratification (based on histological tumor size > 30 mm, vertical margin, venous invasion, and submucosal invasion ≥ 500 µm (SM2)) [23], and EGC with (N+) and without (N0) lymph node metastases were compared. The date of diagnosis of gastric cancer was defined as the date of the upper endoscopy where the malignant biopsy was collected.

### 2.3. Data Collection and Variables

Existing information in the database including age, sex, anatomic location of the tumor, pretreatment disease stage on computer tomography (CT) (UICC 7th edition [24]), final histopathological TNM staging, and Lauren histological type [3] was registered. For patients with EGC, additional information was extracted manually from medical records for the purpose of this study. This included indications for upper endoscopy that led to the diagnosis of EGC and findings at the endoscopy, as well as CT and endoscopic ultrasound (EUS) staging, whenever applied. The recurrence of gastric cancer, death, and cause of death (dichotomized as related to gastric cancer or not) during follow-up were recorded. The censoring date was 31 December 2021, allowing a minimum follow-up of 5 years for all patients.

### 2.4. Histopathological and Immunohistochemical Analyses of EGCs

The histopathological specimen obtained during surgical resection for all EGC patients, excluding those treated endoscopically with mucosal resection (n = 5), were re-evaluated for mucosal (M) and submucosal (SM) invasion with subclassification into SM1 (<500 µm) and SM2 (≥500 µm) [25]. Ulceration was defined as an area of damage to the stomach wall extending beyond the lamina propria. Erosion, on the other hand, was a superficial defect to the mucosa not extending to the submucosa. The presence of vascular and lymphatic invasion, tumor size, Laurens classification [3], and degree of differentiation according to the WHO classification [26] were assessed. In cases where depth of growth and infiltration of tumor into arteries, veins, and lymphatic vessels were difficult to evaluate in HE-stained sections, additional stains (such as Elastica van Gieson) and immunohistochemical (IHC) stains (such as Cytokeratin AE1/AE3, ETS family transcription factor ERG (ERG), and D2-40 (podoplanin)) were performed at the discretion of the pathologist (PM). For IHC labelling, 3 µm sections were cut from formalin-fixed paraffin-embedded tissue blocks. Heat-induced epitope retrieval in cell conditioner # 1 (Roche/Ventana, Tuson, AZ, USA) for 64 min was used before incubation with antibodies. Primary antibodies were used against cytokeratin (CK) (dilution 1:100, incubation time 32 min, code M3515 DAKO/Agilient; Santa Clara, CA, USA), and antibodies were used against podoplanin for the visualization of lymphatic vessels (1:100, incubation time 32 min, code M3619, Dako/Agilent). The immunoreactions were visualized using an OptiView DAB IHC Detection Kit (Code 760-700, Ventana/Roche). Double staining with anti-ERG and anti-CK was performed on selected sections to separate neoplastic from non-neoplastic tissue. Anti-ERG (ready-to-use dilution, code 790-4576, Ventana/Roche) was visualized with an OptiView DAB IHC Detection Kit, followed by anti-CK (dilution 1:100, incubation time 16 min, code M3515, DAKO/Agilient) visualized with UltraView Universal Alkaline Phosphatase Red Detection Kit (Code 760-501, Ventana/Roche). The immunolabelling procedures were run using a BenchMark Ultra system (Ventana/Roche) and counterstained with hematoxylin. Elastica van Gieson staining was performed on 5 µm sections using an elastic staining kit (Code 860-005, Ventana/Roche) following the manufacturer’s instructions.

### 2.5. Statistical Analysis

Using SPSS version 29.0.1 (IBM, Armonk, NY, USA), data were analyzed and presented. Continuous variables were summarized by the median (range) and analyzed using a two-sided Mann–Whitney U test. Categorical variables were presented using crosstabulation and analyzed by the chi-square test or Fisher’s exact test depending on sample size. The analyses of annual numbers and proportions of EGC throughout the study period were performed using univariable linear regression. The Kaplan–Meier method was used to determine long-term survival rates, and differences were assessed by the log rank test. *p*-values below 0.05 were considered significant.

### 2.6. Ethics

The gastric cancer projects have been approved by the Regional Committee for Medical and Health Research Ethics (2011/1436 and 2016/2173).

## 3. Results

**Figure 1:**
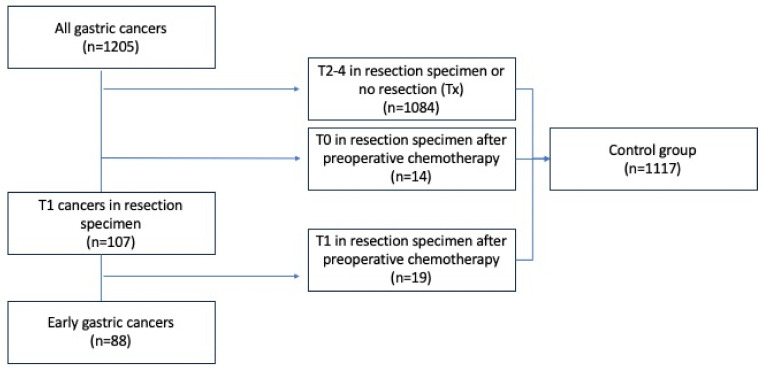
Flow diagram of study population.

A total of 1205 incident gastric cancers were registered in the study period and 88 (7.3%) of these were chemo-naïve EGC (patient flow chart presented in Figure 1). Of these, 83/88 received formal resection and lymphadenectomy, and 5 received endoscopic mucosal resection only. The remaining 1117 (92.7%) patients constituted a control group. Within the control group, 1084 (97.0%) were patients staged with (y)pT2-T4 tumors or patients who did not undergo resection, whereas 14 (1.3%) and 19 (1.7%) patients had ypT0 or ypT1 tumors, respectively, after receiving neoadjuvant chemotherapy.

### 3.1. Annual Number of EGC and Gastric Cancer

**Figure 2:**
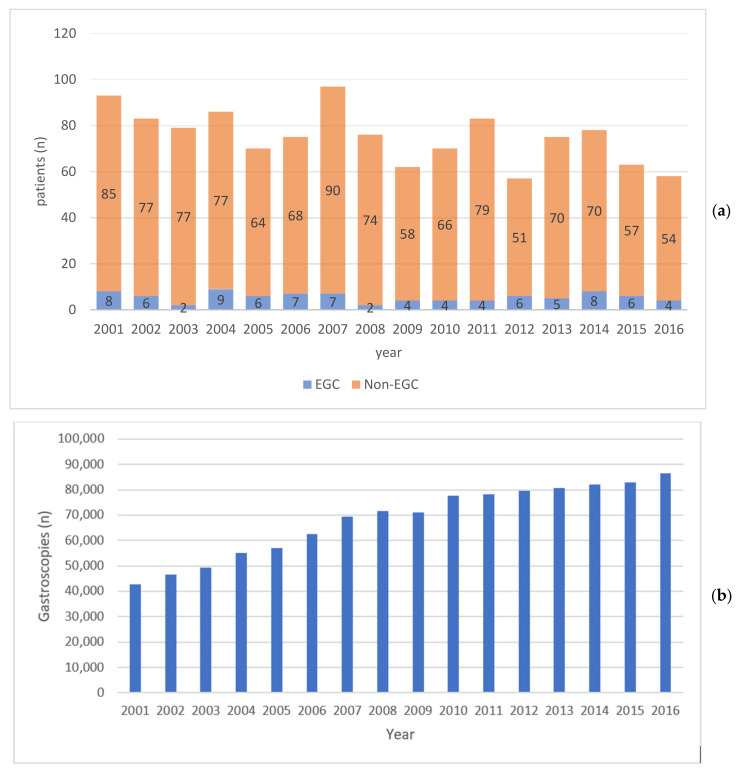
The annual number of early gastric cancers (EGCs) and non-EGCs in Central Norway, 2001–2016 (**a**)**.** The annual number of gastroscopies in Norway, 2001–2016 (**b**).

The annual number of gastric cancers declined during the study period (*p* = 0.010). The annual number of EGCs remained unchanged (*p* = 0.514), however, in such a way that the proportion of EGC/overall number of gastric cancers also remained unchanged (*p* = 0.542) (Figure 2). During the same time period, the annual number of gastroscopies in Norway increased from 42,626 in 2001 to 86,429 in 2016 (*p* < 0.001) (Figure 2b).

### 3.2. Patient Characteristics

The median age of the entire cohort was 75 (21–99) years, and 773 (64.1%) patients were males. Age and sex distribution did not differ significantly between EGCs and controls (Table 1).

### 3.3. Tumor Location and (y)pTNM Stage

The tumor location differed significantly between EGCs and controls (*p* < 0.001), driven by a higher percentage of EGCs confined to either the corpus or antrum, whereas the control group had a higher proportion of cancers with diffuse locations and cancers confined to the cardia (Table 1). While EGCs were pT1 by definition, the control group consisted of a small proportion of ypT0-T1 tumors following neoadjuvant chemotherapy (3.0%) and a large proportion of (y)pT2-T4 cancers (46.6%) (Table 1). Most patients in the control group (50.4%), however, were staged with Tx as they did not undergo resection, either due to metastatic disease (the large majority by far) or because they were considered medically inoperable. The N-stage also differed significantly between EGCs and controls (*p* < 0.001), with 84.1% of EGCs being N0, whereas only 17% of controls were staged as N0, including some that had received pre-operative chemotherapy. Paralleling that the largest proportion of cancers within the control group was assigned a Tx status, an Nx status was correspondingly frequent within this group. None of the EGC patients had known distant metastases compared with 46.1% of controls.

### 3.4. Lauren Classification

The Lauren distribution differed significantly between the EGC and the controls (*p* < 0.001). This was explained by EGCs more often being of the intestinal type compared with controls (73.6% vs. 44.1%) and less frequently being of the diffuse (19.5% vs. 31.2%) and the mixed types (4.6% vs. 11.5%) (Table 1).

### 3.5. Symptoms at Diagnosis and Findings at Upper Endoscopy

Of the patients diagnosed with EGC, epigastric pain (42.0%), GI bleeding or anemia (39.8%), and weight loss or reduced general health (19.3%) were the most common symptoms (Table 2). Vomiting and nausea (17.0%) and symptoms of gastroesophageal reflux (12.5%) were also frequent. Ten (11.4%) patients with EGC did not have symptoms as an indication for upper endoscopy at diagnosis (Table 2), but underwent follow-up of gastric polyps or ulcers, Barrett’s esophagus, or coeliac disease, whereas two patients had an incidental CT finding in the stomach as an indication for upper endoscopy. At the endoscopy, 43 (51.8%) patients had gastric ulcer(s), 40 (48.2%) had a polyp or tumor, and inflamed gastric mucosa was described in 27 (32.5%) patients.

### 3.6. Findings at Pre-Operative EUS and CT

EUS was not part of the standard pre-operative work-up for gastric cancer during the study period and was performed in only 15 (17.0%) EGC patients. Of those, four tumors (26.7%) were staged as T0, five (33.3%) as T1, five (33.3%) as T2, and one (6.7%) as T3. Thirteen (86.7%) tumors were staged with N0 disease and two (13.3) were staged with N1 or more advanced disease (Table 2). Pre-operative CT was performed in 81 (92.0%) of the patients retrospectively concluded to have EGC (Table 2). Of those, sixteen (19.8%) were staged as T0 cancer, two (2.5%) as T1, 12 (14.8%) as T2, five (6.2%) as T3, and two (2.5%) as T4 cancer. However, a majority of 44 (54.3%) patients were radiological Tx. Seventy-one (87.7%) patients were radiologically N0, seven (8.6%) were N1, one (1.2%) was N2, none were staged as N3, and two (2.5%) patients were Nx.

### 3.7. Histopathological Findings and eCura Risk Stratification in N0 and N+ EGC Patients

Eight cases of EGC were N+, with three, four, and one being classified as pN1, pN2, and pN3, respectively. A total of 36 (43.9%) of the tumors were poorly differentiated, 44 (53.7%) were moderately differentiated, and none were well differentiated (Table 3). A total of 39 (48.1%) of the EGCs had a maximal invasion classified as SM2, 28 (34.5%) had maximal invasion classified as M, and 13 (16.0%) were classified as SM1 (Table 3). The median SM depth was 2000 µm (0–9000), and the median tumor size was 20 mm (4–90). A total of 32 (39.0%) patients had no histological ulcers or erosions, whereas 30 (36.6%) had an erosion and 17 (20.7%) had an ulceration. According to the eCura [23] risk stratification, 53 (64.6%), 16 (19.5%), and 10 (12.2%) patients were categorized as low, intermediate, and high risk for lymph node metastases, respectively. Notably, five (9.4%) of the patients that were classified as low risk by eCura had N+ surgical specimens. In a post hoc analysis of eCura low-risk patients, exclusion of patients with histological ulceration or SM2 left a group of 36 patients with N0 surgical specimens.

### 3.8. Recurrence of Cancer and Cause of Death in EGC Patients

Recurrence of cancer was diagnosed in seven (8.1%) patients after a median time of 29 (14–124) months from the EGC diagnosis (Table 4). Among these seven patients, two and one patients, respectively, were classified with pN2 and pN3. A total of 55 (62.5%) EGC patients died during the follow-up period. Of these, forty-three (78.2%) were deaths unrelated to EGC, seven (12.7%) died of cancer recurrence, and five (9.1%) died of postoperative complications. Of the 22 patients who died within five years from diagnosis, 13 (59.1%) died of causes unrelated to EGC, 4 (18.2%) died of cancer recurrence, and 5 (22.7%) died of postoperative complications.

### 3.9. Overall Survival in Patients with EGC Patients

**Figure 3:**
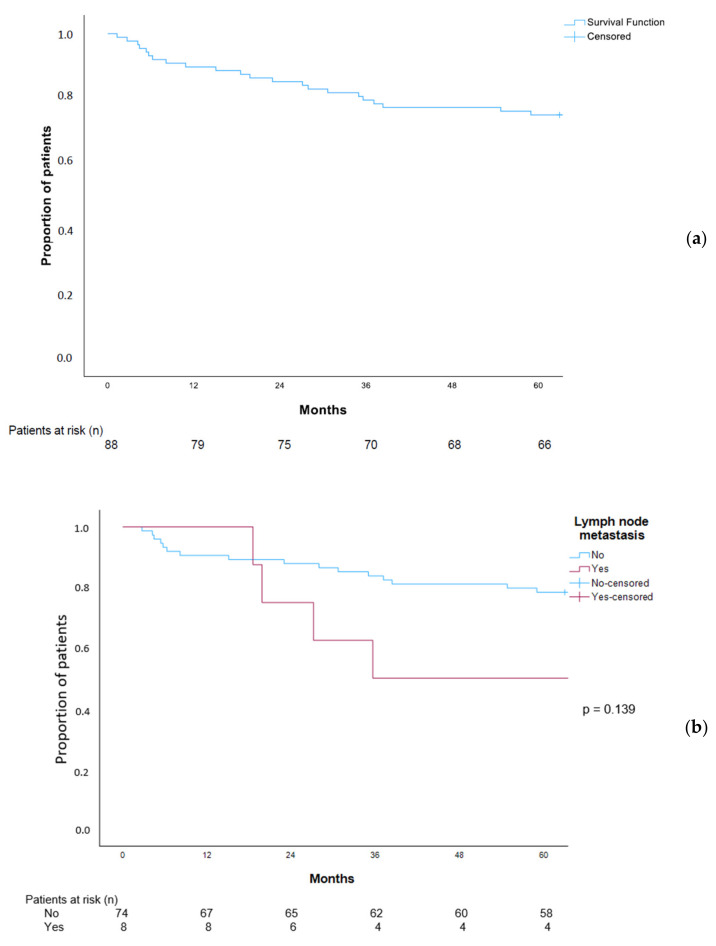

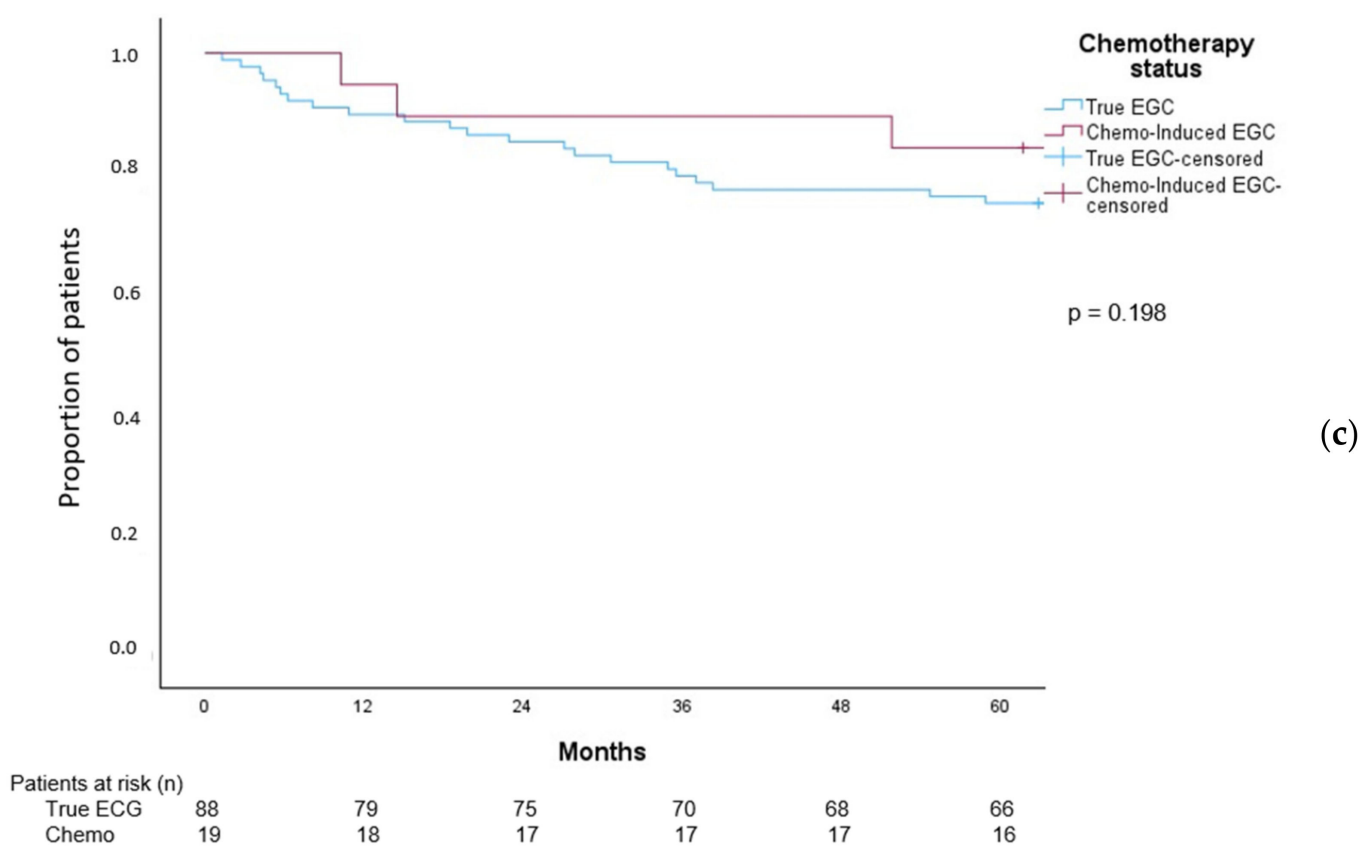
Overall survival in patients with early gastric cancer (EGC) in Central Norway 2001–2016 (**a**) in patients with EGC N0 versus EGC with N+ (**b**) and in chemo-naïve (true) EGC versus chemotherapy-induced EGC (**c**).

The median overall survival for patients with EGC was 117.1 months (95% CI 99.8–134.3), and the 5-year overall survival was 75% (Figure 3a). The median overall survival of EGC N0 patients was 120.7 months (95% CI 100.9–140.6) compared with 35.5 months (95% CI 0.0–100.1) in patients with N+ status (Figure 3b). Although the survival curves visually diverge, the numbers were small and the overall survival did not differ significantly between the groups, log rank *p* = 0.139. Furthermore, the overall survival for patients with chemo-naïve (true) EGC did not differ significantly from that of patients with chemo-induced EGC (ypT0/ypT1), Figure 3c, log rank test, *p* = 0.198.

## 4. Discussion

### 4.1. EGC and GC per Year

Number of gastric cancers in Central Norway decreased steadily during the study period, consistent with numerous other reports of declining rates in Western populations [27]. However, the annual number of EGCs and the relative proportion of EGCs in Central Norway did not significantly change. This was observed despite a doubling in the annual number of upper endoscopies performed per year in Norway during the study period, suggesting that the increase in upper endoscopies did not contribute to earlier diagnosis of gastric cancer. Worldwide, the relative proportions of EGCs vary considerably [9]. The nation-wide screening program in Japan has improved the detection of early-stage disease, and >50% of gastric cancers are EGCs [15,28]. The inclusion of intramucosal neoplasia in the definition of EGC in Japan, however, must be kept in mind. In Western countries, the proportion of EGC has previously been reported to be 5–10% of all gastric cancers [29], consistent with the 7.3% in our cohort. The improved quality of upper endoscopies to reduce the frequency of cancers missed at upper endoscopy [30,31,32], as well as the identification of sub-populations that are at higher risk even in low- to intermediate-risk populations [10], has gained attention in an effort to obtain earlier diagnosis. A recent study from the USA reported an increasing incidence of EGC and a proportion of EGC above 30% [33].

### 4.2. EGC Patient and Tumor Characteristics

Patients with EGC had a median age of 77 years, and 69% were males, which did not significantly differ from the control group. Our EGC patients were, however, significantly older than the 62–63 years, and the proportion of male patients was lower than the 45–54%, often reported in other Western cohorts [34,35]. Although patients with histological T0-T1 after neoadjuvant chemotherapy were excluded from the current EGC cohort, this involved a minor proportion of the patients diagnosed after chemotherapy was introduced in 2007, and the markedly higher age in our cohort did necessarily affect overall survival. A larger proportion of EGCs were of the Lauren intestinal type compared with the controls (73.6% and 44.1% respectively), whereas a similar Lauren distribution has been reported in other studies [36]. The phenomenon may be explained by the fact that the diffuse-type tumors are frequently flat or depressed lesions and are hence challenging to detect during endoscopy [37]. Correspondingly, we recently reported that cancers missed during upper endoscopy were more frequently of the Lauren diffuse type [31].

### 4.3. EGC, Lymph Node Metastases, and Distant Metastases

We found that 9.2% of surgically resected EGC patients were staged with N+ disease. The frequency of N+ among EGCs is generally reported to be 10–15% [35], whereas some have reported a proportion as high as 20% [38,39,40]. To some surprise, of the eight patients with N+ status, four were staged with N2 disease and one was staged with N3 disease, with a correspondingly grave prognosis. None of our EGC patients had evidence of distant metastatic disease at the time of diagnosis. EGC with distant metastases is rare, with a reported incidence of 0.14% in all gastric cancers and 0.37% in early gastric cancers [41].

Ulceration within a lesion is a well-recognized risk factor for N+ [16,42,43,44]. In addition, tumor size, infiltration depth, lymphatic and venous invasion, and low differentiation have also been reported as risk factors for N+ in EGC [15,18,45]. In our relatively small cohort containing only eight N+ patients, these sub-analyses did not reach statistical significance. Patients with N+ had a tumor invasion depth of M, SM1, or SM2 at frequencies similar to previous reports [46,47]. The eCura risk score has recently been proposed to identify patients who after ESD of EGC should be considered for subsequent formal surgical resection. Patients in the eCura low risk category were originally reported to have a cancer specific 5-year survival of 99.6% [48], suggesting a very low frequency of N+ disease. In our cohort, a relatively high proportion (9.4%) of the patients with low risk were N+. Two other Western case series found that 2.9% [34] and 13.6% [35] of patients fulfilling expanded ESD criteria [49] had N+ disease in surgical specimens, and rescue surgery should be considered in patients with low risk of postoperative mortality. In a subsequent post hoc analysis, however, we found that when excluding patients with either histological ulceration or SM2 invasion, none of the remaining 48 low-risk patients had N+ disease. If we apply these criteria, ESD could be a curative treatment in 54.5% (44 of 88) of our EGC patients. The significance of ulceration and SM depth as risk factors for N+ has been emphasized in the original and expanded Japanese criteria for curative ESD treatment [15,16].

### 4.4. EGC and Imaging

Although the accuracy of single-detector CT (69–84%) and multi-detector CT (80–89%) for gastric cancer staging overall has been reported [50], the ability of CT to separate early T-stages is limited [51,52], and in our cohort, the T-stage was set as Tx in the majority of cases and correctly in less than 3%. Similarly, EUS analysis provided accurate T-staging in one-third of the patients who underwent the examination. This is significantly lower than the accuracy of EUS examinations performed in Japan, where the accuracy of depth growth is reported to be 65–86% [53], possibly reflecting the infrequent use and lack of experience with EUS at our institution during the study period.

### 4.5. Survival in EGC Patients

In the current study, the 5-year overall survival was 75% in the EGC cohort and the median survival was 117.1 months (95% CI 99.8–134.3). This is on par with the 5-year survival from EGC in other Western countries reported to be within the range 69–82% [54]. In our cohort, seven (8.0%) patients died of gastric cancer during follow-up, of whom three were classified as pN2 or pN3, reflecting the grave prognosis of more advanced N+ disease. Others have reported no cancer-related mortality after follow-up with Western EGC patients treated with ESD; however, the median and minimum follow-up time was not stated [55]. Notably, three cancer deaths in our cohort occurred more than five years from diagnosis. This demonstrates that 5-year survival is an insufficient measure of disease-specific survival in EGC patients. Others have observed that 23% to 33% of EGC recurrences were detected more than five years after the primary treatment [56,57,58]. There was no statistically significant difference in survival for EGC patients with and without lymph node metastasis, likely due to a type II error, it should be observed that the survival curves diverge Figure 3b, but that numbers are small.

Five EGC patients (5.7%) died of postoperative complications, which is a high proportion and particularly unfortunate for a cancer entity with a relatively good prognosis. Many Western and Asian centers report ESD-related mortality below 1% [55], also in patients > 80 years of age [59,60]. Keeping a median age of 77 years for the study cohort in mind, a high proportion of EGC patients may benefit from primary ESD for risk stratification followed by formal surgery in selected patients [19,22]. Finally, we also compared survival in EGC patients with patients with ypT0-T1, i.e., after receiving pre-operative chemotherapy, where most patients were likely to have been down-staged before surgery. There was no indication that patients with chemotherapy-induced ypT0-1 had a worse prognosis, that is, the down-staging was not merely a cosmetic finding but seemed to confer a true survival benefit. Other studies have reported a similar prognosis of ypT1-2 cancers compared with pT1-2 cancers treated by upfront surgery [61,62], which parallels recently published observations even for stage II and III disease in the current control group [63].

### 4.6. Strengths and Limitations

This Western population-based cohort of EGC patients is based on data from high-quality patient registries, allowing trends in EGC over time to be analyzed. The project had access to surgical specimens for histopathological re-evaluation and a near-complete follow-up of all patients, which allowed recurrence and death during follow-up to be recorded. The study was limited by its retrospective nature and a low number of EGC patients compared with Eastern cohorts, which prevented further sub-analyses. CT images could not be re-valuated, and the study was not designed to assess the true value of pre-operative imaging.

## 5. Conclusions

The population-based proportion of EGCs in Central Norway was 7.3% and remained unchanged during the period of 2001–2016, despite the number of upper endoscopies performed being more than doubled during the same period. EGCs were associated with location to the corpus and antrum and were more frequently of the Lauren intestinal type. EGCs were often staged incorrectly during CT and EUS. In this historic cohort with formal gastric resection for EGC, 9.0% had pN+ disease. Retrospective risk assessment by eCura score alone was insufficient to identify patients with pN0. The overall survival for patients with EGC in this study was on par with other publications reviewing survival in EGC patients.

## Figures and Tables

**Table 1:** Patient demographics, tumor location, (y)pTNM stage, and Lauren classification of gastric cancers in Central Norway during 2001–2016 (n = 1205).

Variable	Entire Cohort	EGC	Non-EGC	*p*-Value
Patients, n (%)	1205	88 (7.3)	1117 (92.7)	
Age at diagnosis, years				0.137
Median (range)	75 (21−99)	77 (43−91)	74 (21−99)	
Sex, n (%)				0.294
Male	773 (64.1)	61 (69.3)	712 (63.7)	
Cancer location, n (%)				<0.001
Cardia	337 (28.0)	13 (14.8)	324 (29.0)	
Corpus	328 (27.2)	32 (36.4)	296 (26.5)	
Antrum	392 (32.5)	43 (48.9)	349 (31.2)	
Diffuse	142 (11.8)	0 (0.0)	142 (12.7)	
Not recorded	6 (0.5)	0 (0.0)	6 (0.5)	
(y)pT stage, n (%)				
T0	14 (1.2)	-	14 (1.3)	
T1	107 (8.9)	88 (100.0)	19 (1.7)	
T2	55 (4.6)	-	55 (4.9)	
T3	178 (14.8)	-	178 (16.0)	
T4a	204 (17.0)	-	204 (18.3)	
T4b	82 (6.8)	-	82 (7.4)	
Tx	560 (46.7)	-	560 (50.4)	
(y)pN stage, n (%)				<0.001
N0	268 (22.4)	74 (84.1)	189 (17.0)	
N1	101 (8.4)	3 (3.4)	98 (8.8)	
N2	106 (8.9)	4 (4.5)	102 (9.2)	
N3	120 (10.0)	1 (1.1)	119 (10.7)	
Nx	602 (50.3)	6 (6.8)	601 (54.2)	
(y)pM stage, n (%)				<0.001
M0	605 (50.2)	88 (100.0)	520 (46.6)	
M1	515 (42.7)	0 (0.0)	515 (46.1)	
Mx	85 (7.1)	0 (0.0)	82 (7.3)	
Lauren classification, n (%)				<0.001
Diffuse	365 (30.3)	17 (19.5)	348 (31.2)	
Intestinal	557 (46.3)	64 (73.6)	493 (44.1)	
Mixed diffuse/intestinal	132 (11.0)	4 (4.6)	128 (11.5)	
Cancer NUD/No biopsy	150 (12.5)	2 (2.3)	148 (13.2)	

EGC: Early gastric cancer; NUD: non-numerical unstructured data.

**Table 2:** Symptoms at diagnosis, findings at upper endoscopy, and pre-operative imaging in EGCs in Central Norway during 2001–2016.

Variable	
Symptoms at time of diagnosis (total n = 88), n (%)	
No symptoms	10 (11.4)
Dysphagia	8 (9.1)
Epigastric pain	37 (42.0)
GI-bleeding/anemia	35 (39.8)
Vomiting/nausea	15 (17.0)
Acid reflux	11 (12.5)
Weight loss/reduced general health	17 (19.3)
No information	2 (2.3)
Findings at upper endoscopy (total n = 83), n (%)	
Inflammation	27 (32.5)
Ulcer	43 (51.8)
Polyp or tumor	40 (48.2)
EUS T-staging (total n = 15), n (%)	
T0	4 (26.7)
T1	5 (33.3)
T2	5 (33.3)
T3	1 (6.7)
T4	0 (0.0)
EUS N-staging	
N0	13 (86.7)
N1 or more	2 (13.3)
Pre-operative imaging	
CT	81 (92.0)
Ultrasound	2 (2.3)
No imaging	5 (5.7)
CT T-staging, n (%)	
T0	16 (19.8)
T1	2 (2.5)
T2	12 (14.8)
T3	5 (6.2)
T4	2 (2.5)
Tx	44 (54.3)
CT N-staging, n (%)	
N0	71 (87.7)
N1	7 (8.6)
N2	1 (1.2)
N3	0 (0.0)
Nx	2 (2.5)
CT M-staging, n (%)	
M0	78 (96.3)
M1	0 (0.0)
Mx	3 (3.7)

GI: Gastrointestinal; EUS: endoscopic ultrasound; CT: computer tomography; T: tumor; N: node; M: metastasis.

**Table 3:** Histopathological findings in surgically resected early gastric cancer (EGC) with a defined N0 or N+ status (n = 82) in Central Norway, 2001–2016.

Variable	Total	EGC N0	EGC N+	*p*-Value
Patients, n (%)	82 (100.0)	74 (90.8)	8 (9.2)	
Differentiation, n (%)				0.558
Poor	36 (43.9)	31 (41.9)	5 (62.5)	
Moderately	44 (53.7)	41 (55.4)	3 (37.5)	
Uncertain	2 (2.4)	2 (2.7)	0 (0.0)	
Lauren, n (%)				0.272
Diffuse	17 (20.7)	15 (20.3)	2 (25.0)	
Intestinal	59 (72.0)	54 (73.0)	5 (62.5)	
Mixed	4 (4.9)	4 (5.4)	0 (0.0)	
Cancer NUD/no biopsy	2 (2.4)	1 (1.4)	1 (12.5)	
Depth of invasion, n (%)				0.879
M	28 (34.5)	26 (35.6)	2 (25.0)	
SM1	13 (16.0)	12 (16.4)	1 (12.5)	
SM2	39 (48.1)	34 (46.6)	5 (62.5)	
Uncertain	2 (1.2)	2 (1.4)	0 (0.0)	
Tumor size, mm				
Median, (range) ^a^	20 (4–90)	20 (4–90)	22.5 (10–60)	0.401
SM depth, µm				
Median, (range)	2000 (0–9000)	2000 (0–9000)	2000 (400–2800)	0.794
Ulceration, n (%)				
None	32 (39.0)	30 (39.2)	2 (25.0)	0.145
Erosion	30 (36.6)	28 (37.8)	2 (25.0)	
Ulceration	17 (20.7)	13 (17.6)	4 (50.0)	
Uncertain	3 (3.7)	3 (4.1)	0 (0.0)	
Vascular invasion, n (%)				
None	51 (63.3)	47 (63.5)	4 (50.0)	
Lymphatic	17 (21.8)	15 (21.4)	2 (25.0)	
Arterial	0 (0.0)	0 (0.0)	0 (0.0)	
Venous	12 (15.4)	10 (14.3)	2 (25.0)	
Uncertain	2 (2.4)	2 (2.7)	0 (0.0)	
eCura scores, n (%)				
Low risk	53 (64.6)	48 (64.9)	5 (62.5)	
Intermediate risk	16 (19.5)	14 (18.9)	2 (25.0)	
High risk	10 (12.2)	9 (12.2)	1 (12.5)	
Incomplete data	3 (3.7)	3 (4.1)	0 (0.0)	

GI: Gastrointestinal; NUD: non-numerical unstructured data; Depth of invasion; M: mucosal; SM: submucosal. ^a^ Two patients with uncertain tumor size.

**Table 4:** Cancer recurrence and cause of death in early gastric cancer patients in Central Norway, 2001–2016.

Variable	
Recurrence (available for n = 86), n (%)	7 (8.1)
Time to recurrence, months. Median (range).	29 (14–124)
Died during follow-up, n (%)	55 (62.5)
Cause of death, n (%)	
Cancer recurrence	7 (12.7)
Not cancer-related	43 (78.2)
Postoperative complications	5 (9.1)
Cause of death within 5 years, n (%)	
Cancer recurrence	4 (18.2)
Not cancer related	13 (59.1)
Postoperative complications	5 (22.7)

## Data Availability

The project data cannot be shared according to regulations given by the Regional Committee for Medical and Health Research Ethics. The study has used data from the Cancer Registry of Norway. The interpretation and reporting of these data are the sole responsibility of the authors, and no endorsement by the Cancer Registry of Norway is intended, nor should it be inferred.

## Abbreviations

CI	Confidence interval
CT	computed tomography
eCura	Endoscopic curability
EGC	Early gastric cancer
ESD	Endoscopic submucosal dissection
EUS	Endoscopic ultrasound
GI	Gastrointestinal
IHC	Immunohistochemical
LNM	Lymph node metastases
NPR	Norwegian patient registry
NUD	Non-numerical unstructured data
SM	Submucosal
